# Oligohydramnios in Women with Preterm Prelabor Rupture of Membranes and Adverse Pregnancy and Neonatal Outcomes

**DOI:** 10.1371/journal.pone.0105882

**Published:** 2014-08-29

**Authors:** Marian Kacerovsky, Ivana Musilova, Ctirad Andrys, Marcela Drahosova, Helena Hornychova, Adam Rezac, Milan Kostal, Bo Jacobsson

**Affiliations:** 1 Department of Obstetrics and Gynecology, Charles University in Prague, Faculty of Medicine in Hradec Kralove, University Hospital in Hradec Kralove, Hradec Kralove, Czech Republic; 2 Biomedical Research Center, University Hospital in Hradec Kralove, Hradec Kralove, Czech Republic; 3 Department of Clinical Immunology and Allergy, Charles University in Prague, Faculty of Medicine in Hradec Kralove, University Hospital in Hradec Kralove, Hradec Kralove, Czech Republic; 4 Fingerland's Department of Pathology, Charles University in Prague, Faculty of Medicine in Hradec Kralove, University Hospital in Hradec Kralove, Hradec Kralove, Czech Republic; 5 Department of Obstetrics and Gynecology, University Hospital in Pardubice, Pardubice, Czech Republic; 6 Department of Obstetrics and Gynecology, Sahlgrenska University Hospital, Gothenburg, Sweden; 7 Department of Public Health, Oslo University, Oslo, Norway; The Ohio State Unversity, United States of America

## Abstract

**Objective:**

To determine the association between the presence of oligohydramnios, determined as an amniotic fluid index ≤ 5 cm and the intra-amniotic inflammatory response, fetal inflammatory response and neonatal outcomes in actively managed preterm prelabor rupture of membranes (PPROM).

**Methods:**

Women with singleton pregnancies complicated by PPROM at a gestational age of between 24+0 and 36+6 weeks were included in the study. Ultrasound assessments of the amniotic fluid index and evaluation of the amniotic fluid interleukin (IL)-6 levels were performed at admission. The umbilical cord blood IL-6 levels were evaluated after delivery.

**Results:**

In total, 74 women were included. The women with oligohydramnios did not have different amniotic fluid IL-6 levels [with oligohydramnios: median 342 pg/mL, interquartile range (IQR) 110-1809 vs. without oligohydramnios: median 256 pg/mL, IQR 122–748; *p* = 0.71] or umbilical cord blood IL-6 levels (with oligohydramnios: median 8.2 pg/mL, IQR 3.8–146.9 vs. without oligohydramnios: median 5.9 pg/mL, IQR 2.1–27.9; *p* = 0.14) than those without oligohydramnios. No association between oligohydramnios and neonatal morbidity was found. A correlation between the amniotic fluid index and the interval from rupture of membranes to amniocentesis was observed (rho = −0.34; *p* = 0.003).

**Conclusion:**

The presence of oligohydramnios is not associated with an adverse outcome in actively managed PPROM in singleton pregnancies in the absence of other complications.

## Introduction

Preterm prelabor rupture of membranes (PPROM) is a serious complication of pregnancy, occurring in approximately 3–4% of all deliveries [1], [2]. PPROM is defined as rupture of the fetal membranes with leakage of amniotic fluid occurring before the onset of regular uterine activity prior to 37 completed weeks of gestation [1].

PPROM is complicated by microbial invasion of the amniotic cavity (MIAC) and histological chorioamnionitis (HCA) in approximately 30% and 60% of PPROM cases, respectively [3], [4]. The presence of these conditions is followed by an intra-amniotic and fetal inflammatory response that is associated with an adverse neonatal outcome [3]–[7]. Early identification of these complications appears to be crucial for the optimal management of PPROM and improved neonatal outcomes.

In addition to the maternal circulation, amniotic and cervicovaginal fluid markers, ultrasound evaluation might be a potential non-invasive tool for identifying women with PPROM who are at high risk of infectious and inflammatory complications [3], [4], [8]–[12]. Previous studies have suggested potential ultrasound markers such as the fetal thymus, splenic vein pulsation and presence of oligohydramnios after PPROM [11]–[14]. Some results concerning the association between oligohydramnios and adverse outcomes appear to be conflicting [13], [15]–[17]. The policy of active management of women with PPROM in the Czech Republic provides a unique opportunity for an evaluation of the amniotic fluid, placenta and umbilical cord blood, with limited time discrepancies between the sampling of the different compartments.

The major aim of this study was to evaluate the association between oligohydramnios after PPROM and the presence of MIAC and HCA and the intra-amniotic and fetal inflammatory response. An additional aim was to evaluate the association between oligohydramnios and short-term neonatal morbidity.

## Methods

### Sample collection

Between February 2012 and August 2013, a prospective cohort study was conducted on pregnant women between 24+0 and 36+6 gestational weeks who were admitted to the Department of Obstetrics and Gynecology of the University Hospital in Hradec Kralove, Czech Republic. Pregnant women with singleton pregnancies, PPROM and maternal age > 18 years were invited to participate in the study. The exclusion criteria included women with gestational hypertension, preeclampsia, fetuses with an estimated weight below the 10^th^ percentile, the presence of congenital or chromosomal fetal abnormalities, gestational or pre-gestational diabetes and signs of fetal hypoxia.

The gestational age was confirmed by ultrasound fetal biometry in the first trimester. PPROM was defined as amniotic fluid leakage preceding the onset of labor by at least 2 h. PPROM was diagnosed visually using a sterile speculum examination to confirm the pooling of amniotic fluid in the vagina and was confirmed by a positive test for the presence of insulin-like growth factor–binding protein-1 (ACTIM PROM test; Medix Biochemica, Kauniainen, Finland) in the vaginal fluid, when necessary [18]. Interval from rupture of membranes to amniocentesis was defined as a time interval (hours) between an initial rupture of membranes with leakage of amniotic fluid based on the woman's self report and the amniocentesis after the admission.

Ultrasound-guided transabdominal amniocentesis was performed upon admission, prior to the administration of antibiotics. The amniotic fluid samples were immediately divided into three polypropylene tubes. The first and second tubes, containing non-centrifuged samples, were immediately transported to the microbiology laboratory, where the first tube was used for polymerase chain reaction (PCR) testing for *Ureaplasma* spp., *Mycoplasma hominis* and *Chlamydia trachomatis* and the second tube was used for aerobic and anaerobic bacterial culture. The third tube was centrifuged for 15 min at 2000×*g* to remove the cells and debris, divided into aliquots and stored at −70°C until analysis.

The management of PPROM in the Czech Republic is active (except < 28 gestational weeks); induction of labor is initiated or an elective cesarean section is performed no later than 72 h after the rupture of the membranes, depending on the gestational age of the pregnancy, the fetal status, the maternal serum levels of C-reactive protein and cervicovaginal streptococcus β colonization. A complete course of antenatal steroids, 14 mg of betamethasone (two ampoules) by intramuscular injection in two doses given 24 h apart, are administered before 34 completed weeks of gestation. Tocolytics are administered for 48 h during the corticosteroid course, and all women with PPROM receive antibiotics. Endocervical administration of dinoprostone or intravenous administration of oxytocin was used for the induction of labor. The decision regarding the technique of induction was made by the lead physicians of the labor and delivery ward based on the Bishop score and other clinical conditions.

After the delivery of the neonates and prior to the delivery of the placenta, umbilical cord blood samples were obtained by venipuncture from the clamped umbilical cords using a vacutainer blood collecting system. The umbilical cord blood samples were centrifuged and aliquoted, and the supernatant was stored at −70°C until analysis.

The study was approved by the University Hospital Hradec Kralove review board committee (March 19, 2008; No. 200804 SO1P), and written informed consent was obtained from all of the participants.

### Sonographic evaluation of the presence of oligohydramnios

Ultrasound evaluation of the amniotic fluid volume was performed at the time of admission and before the amniocentesis and administration of corticosteroids, tocolytics and antibiotics. The methodology of the measurement of the amniotic fluid index was described by Phelan et al. [19]. Color Doppler was used to identify the umbilical cord free pockets of amniotic fluid. Oligohydramnios was defined as an amniotic fluid volume of ≤ 5.0 cm. The sonographic evaluation was performed using an Aplio SSA-77A (Toshiba, Japan) with a convex transabdominal probe at 3.5–7 MHz. Two experienced sonographers performed the measurements. Intraobserver intra-class correlation coefficients (ICC) for the measurement of amniotic fluid index, assessed from two measurements of amniotic fluid index within 10 minutes in 10 women with PPROM by each examiner, were 0.93 [95% confidence interval (CI): 0.74–0.98] and 0.96 (CI: 0.85–0.99) for examiner M.K. and I.M., respectively. The interobserver ICC, assessed from the measurements of amniotic fluid index of 10 women with PPROM, was 0.96 (CI: 0.89–0.99).

### Diagnosis of MIAC

MIAC was defined as a positive PCR for genital mycoplasmas (*Ureaplasma parvum, Ureaplasma urealyticum* and *Mycoplasma hominis*) and/or *Chlamydia trachomatis* and/or as the growth of any bacteria in the amniotic fluid except coagulase-negative *Staphylococcus epidermidis*, which was considered a skin contaminant [20].

### Diagnosis of HCA

The degree of neutrophil infiltration was evaluated separately in the free membranes (amnion and chorion-decidua), in the chorionic plate and in the umbilical cord, based on the criteria provided by Salafia [21]. A diagnosis of HCA was based on histological grades of 3–4 for the chorion-decidua (the multiple or confluent foci of at least 5–20 neutrophils), 3–4 for the chorionic plate (at least a few neutrophils present in the connective tissue or the chorionic plate), 1–4 for the umbilical cord (any neutrophils present in the umbilical cord) and/or 1–4 for the amnion (at least one focus of at least five neutrophils) [21]. Histological grades of 1–4 for the umbilical cord were considered to indicate the presence of funisitis [21]. The histopathological examinations were performed by a single pathologist who was blinded to the clinical status of the women.

### Measurement of the intra-amniotic inflammatory response

The intensity of the intra-amniotic inflammatory response was measured by determining the IL-6 concentration in the amniotic fluid [22], [23]. The IL-6 levels were assessed by a Millenia QuickLine IL-6 lateral flow immunoassay using a Millenia POCScan Reader (R&D Systems Inc., Minneapolis, MN, USA). The measurement range was 50–10000 pg/mL. The sensitivity of the test was less than 50 pg/mL.

### Measurement of the fetal inflammatory response

The intensity of the fetal inflammatory response was measured by determining the IL-6 concentration in the umbilical cord blood. The IL-6 concentration in the umbilical cord blood samples was assessed by ELISA (R&D Systems Inc., Minneapolis, MN, USA). The sensitivity of the test was less than 0.70 pg/mL, and the interassay and intra-assay coefficients were less than 10%. The major reason for using IL-6 to indicate the fetal inflammatory response was that IL-6 umbilical cord blood levels are not expected to change as gestation progresses [24].

### Diagnosis of severe neonatal morbidity

The data on the neonatal morbidity and mortality were retrieved from the maternal and neonatal medical records by three investigators (MK, IM, and AR). We defined severe neonatal morbidity as a condition consisting of respiratory distress syndrome (the presence of two or more of the following criteria: evidence of respiratory compromise, a persistent oxygen requirement for more than 24 h, administration of an exogenous surfactant and radiographic evidence of hyaline membrane disease); bronchopulmonary dysplasia (infant oxygen requirement at 28 days of age); intraventricular hemorrhage (diagnosis performed by transfontanel cerebral sonography according to the procedure of Papile et al.[25]); retinopathy of prematurity (identified using retinoscopy); necrotizing enterocolitis (radiological finding of intramural gas or free intra-abdominal gas); and an early (during the first 72 h of life) and late (between 4 and 120 days of age) onset of sepsis (proven by bacterial culture of clinical highly suspected sepsis). The pediatricians were blinded to the results of the ultrasound evaluation of the amniotic fluid index.

### Statistical analysis

The continuous variables were compared using an unpaired t-test, with the values presented as the means ± SD, or using the non-parametric Mann-Whitney *U* test, with the values presented as medians [interquartile range (IQR)]. The normality of the data was tested using the D'Agostino-Pearson omnibus normality test and the Shapiro-Wilk test. The categorical variables were compared using Fisher's exact test, and the values are presented as numbers (%). Spearman's rank correlation test was used for the analysis of the correlation between the continuous variables. ICC was used to assess intra- and interobserver reliability. All of the *p*-values are from two-sided tests, and all of the statistical analyses were performed using GraphPad Prism 5.03 for Mac OS X (GraphPad Software, La Jolla, CA, USA), SPSS 19.0 for Mac OS W (SPSS Inc., Chicago, IL, USA), and MedCalc 12.3.0 (MedCalc Software, Belgium).

## Results

### Demographic and clinical characteristics of the study population

A total of 79 women with PPROM occurring from 24+0 to 36+6 weeks of gestational age were recruited. Five women could not be included in the study for the following reasons: amniocentesis was not possible (n = 1); the placental histopathological assessments were not available (n = 2); or the umbilical cord blood was missing (n = 2). In the remaining 74 women, the overall rate of MIAC was 28% (21/74), and HCA was found in 66% (49/74) of the women. Fifty-four percent (40/74) of the women had oligohydramnios at admission, and 46% (34/74) of the women were without ultrasound signs of oligohydramnios. The women with oligohydramnios exhibited a shorter interval from rupture of membranes to the amniocentesis, a lower amniotic fluid index and a higher rate of cesarean section. No differences in the rates of MIAC and HCA between the groups with and without oligohydramnios were revealed (Table 1).

**Table 1:** Maternal and neonatal characteristics in the group of women with preterm prelabor rupture of membranes with respect to the presence and absence of oligohydramnios.

	With oligohydramnios (n = 40)	Without oligohydramnios (n = 34)	*p*-value
Maternal age (years)	31.1±6.9	29.0±5.7	0.23
Primiparous	19 (48%)	15 (44%)	0.82
Pre-pregnancy body mass index	22.4 (16.5–32.7)	22.7 (17.9–34.2)	0.66
Smoking	5 (13%)	9 (26%)	0.15
Gestational age at admission (weeks+days)	32+5 (24+0-36+6)	32+0 (24+1-36+3)	0.84
Gestational age at delivery (weeks+days)	33+1 (24+0-36+6)	32+4 (24+4-36+4)	0.72
Interval from rupture of membranes to amniocentesis (hours)	7 (2–170)	4 (1–72)	**0.02**
Amniotic fluid index (cm)	2.4 (0.5–4.9)	9.2 (5.2–18.0)	**< 0.0001**
Latency from amniocentesis to delivery (hours)	38 (4–151)	43 (4–173)	0.76
Amniotic fluid IL-6 (pg/mL)	342 (50–10000)	256 (50–10000)	0.70
CRP levels at admission (mg/L)	6.3 (1.1–61.1)	6.4 (0.5–68.5)	0.93
WBC count at admission (x10^9^ L)	11.9 (7.4–20.6)	12.3 (7.8–29.0)	0.25
Cephalic presentation	30 (75%)	30 (88%)	0.23
Breach presentation	10 (25%)	4 (12%)	0.23
Microbial invasion of the amniotic cavity	12 (30%)	9 (26%)	0.80
The presence of genital mycoplasmas in the amniotic fluid	9 (22%)	6 (18%)	0.77
Caesarean delivery	21 (52%)	9 (26%)	**0.03**
Birth weight (grams)	1894±712	2006±542	0.45
Histological chorioamnionitis	31 (78%)	22 (65%)	0.30
Funisitis	19 (45%)	10 (29%)	0.15
Apgar score < 7 at 5 min	3 (8%)	0 (0%)	0.27
Apgar score < 7 at 10 min	1 (3%)	0 (0%)	1.00

The continuous variables were compared using an unpaired t-test (values presented as the means ± SD) or the non-parametric Mann-Whitney *U* test [values presented as the medians (range)]. The categorical variables were compared using Fisher's exact test [values presented as numbers (%)].

The statistically significant differences are marked in bold.

Abbreviations:

PPROM preterm prelabor rupture of membranes.

IL-6 interleukin-6.

CRP C-reactive protein.

WBC white blood cells.

### Amniotic fluid IL-6 levels

**Figure 1:**
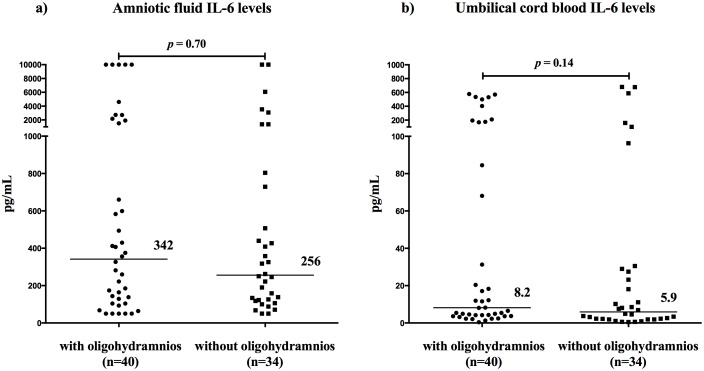
The amniotic fluid (a) and umbilical cord blood (b) interleukin-6 levels with respect to the presence and absence of oligohydramnios. The horizontal bars indicate the median values.

No difference in the amniotic fluid IL-6 levels between the women with and without oligohydramnios was found [with oligohydramnios: median 342 pg/mL (IQR: 110–1809) vs. median 256 pg/mL (IQR: 122–748); *p* = 0.71; see Figure 1)

### Umbilical cord blood IL-6 levels

No differences in the umbilical cord blood IL-6 levels between the women with and without oligohydramnios were identified [median 8.2 pg/ml (IQR: 3.8–147) vs. median 5.9 pg/ml (range: 2.1–27.9); *p* = 0.14; see Figure 1).

### Interval from the rupture of membranes to amniocentesis

**Figure 2:**
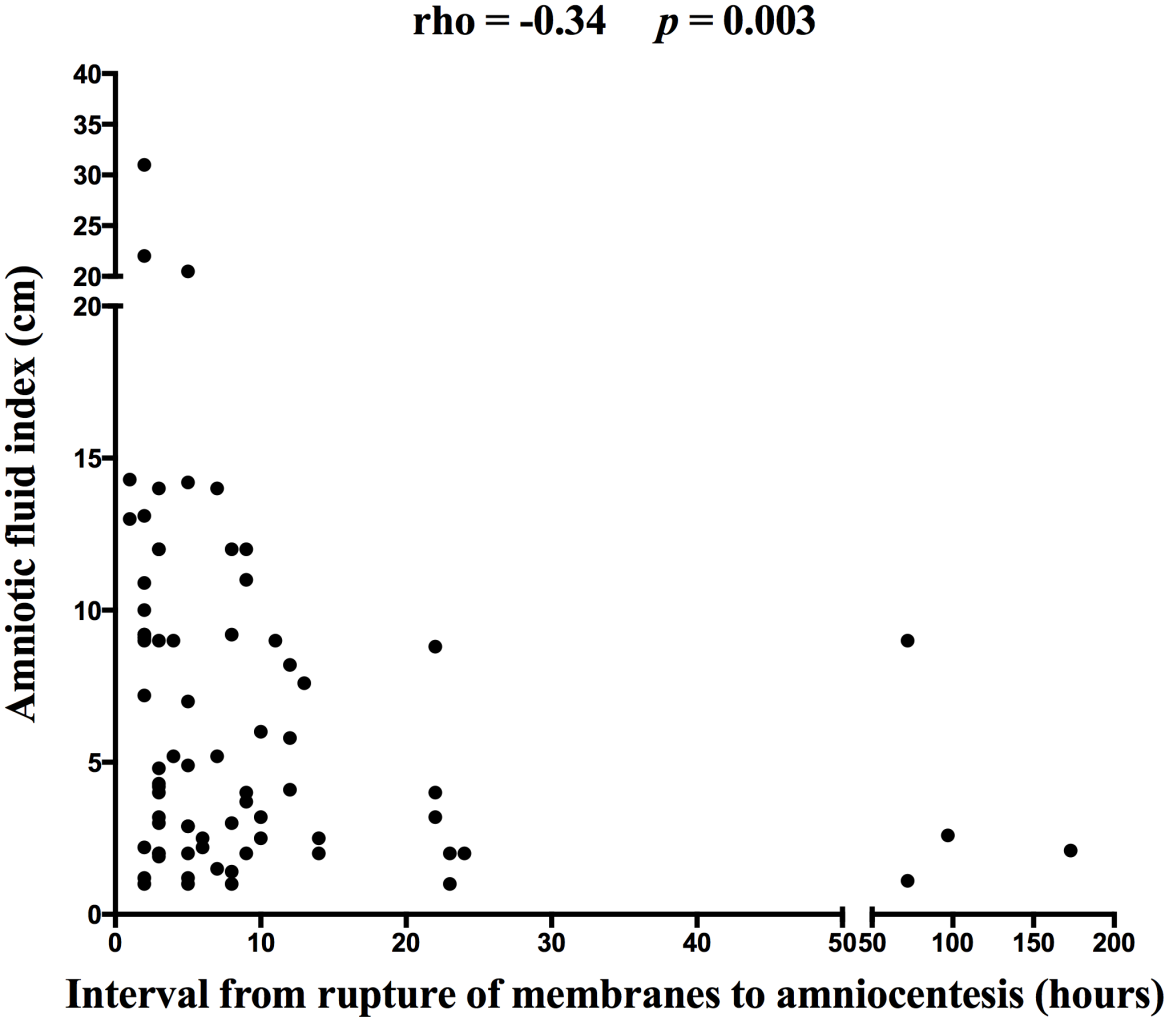
The correlation between the amniotic fluid level determined by the amniotic fluid index (cm) and an interval from PPROM to amniocentesis (hours).

A negative correlation was observed between the amniotic fluid index and the interval from the rupture of membranes to amniocentesis (rho = −0.34; *p* = 0.003; Figure 2) in the crude analysis as well as after adjustment for the gestational age at the time of amniocentesis (*p* = 0.04).

### Neonatal outcome

The association between the presence of oligohydramnios and the selected conditions of severe neonatal morbidity is displayed in Table 2. No significant association was observed.

**Table 2:** The presence and absence of oligohydramnios with respect to the presence of selected conditions of severe neonatal morbidity.

	With oligohydramnios	Without oligohydramnios	*p*-value
	(n = 40)	(n = 34)	
Tracheal intubation	3 (8%)	2 (6%)	1.00
Respiratory distress syndrome	12 (30%)	14 (41%)	0.34
Intraventricular hemorrhage	4 (10%)	9 (26%)	0.08
Intraventricular hemorrhage grade III and IV	1 (3%)	0 (0%)	1.00
Necrotizing enterocolitis	1 (3%)	0 (0%)	1.00
Retinopathy of prematurity	1 (3%)	1 (3%)	1.00
Early-onset sepsis	2 (5%)	1 (3%)	1.00
Late-onset sepsis	1 (3%)	1 (3%)	1.00
Bronchopulmonary dysplasia	4 (10%)	2 (6%)	0.68
Pneumonia	1 (3%)	2 (6%)	0.59
Neonatal death before hospital discharge	3 (8%)	1 (3%)	0.62
Severe neonatal morbidity	18 (45%)	19 (56%)	0.48

The categorical variables were compared using Fisher's exact test [values presented as numbers (%)].

Severe neonatal morbidity was defined as a need for intubation, respiratory distress syndrome, pneumonia, bronchopulmonary dysplasia, retinopathy of prematurity, intraventricular hemorrhage, necrotizing enterocolitis, early-onset sepsis, late-onset sepsis and/or neonatal death before hospital discharge.

## Discussion

The following are the key findings of the study: 1) the presence of oligohydramnios is not associated with higher rates of MIAC and HCA; 2) the presence of oligohydramnios is associated with a longer interval from rupture of membranes to amniocentesis and with a higher rate of cesarean section; 3) the amniotic fluid index negatively correlates with the interval from rupture of membranes to amniocentesis; 4) neither the intra-amniotic nor fetal inflammatory response is affected by the presence of oligohydramnios; 5) there is no association between the presence of oligohydramnios and the neonatal outcome.

Amniotic fluid is responsible for the maintenance of an optimal environment for fetal growth and development [26]. In addition to other functions, amniotic fluid has an antimicrobial property that aids in the protection of the amniotic cavity against microbial invasion [27]. It has been suggested that the presence of oligohydramnios after PPROM reduces this arm of antimicrobial defense and predisposes toward intrauterine infection [13]. Some authors have reported an association between oligohydramnios and MIAC and/or HCA [13], [16], [17], [28], [29]. We found no differences in the MIAC and HCA rates between those with and without oligohydramnios. This finding is in concordance with a study by Mercer et al., which showed no association in the HCA rates and funisitis in the women with and without oligohydramnios measured by the amniotic fluid index and the maximum vertical fluid pocket [15]. We could only hypothesize as to whether an interval from rupture of membranes to amniocentesis (median 5 h) in this study is too short for an ascension of microorganisms to the amniotic cavity because of low levels of antimicrobial peptides resulting from a small amount of residual amniotic fluid.

The evidence indicates that the presence of oligohydramnios in PPROM is related to a shorter latency (interval between rupture of membranes and delivery) compare to PPROM without oligohydramnios [15], [16], [29], [30]. However, there is a lack of information on whether the interval from rupture of membranes to amniocentesis affects the rate of oligohydramnios in PPROM. Only a study by Yoon et al. evaluated the interval from rupture of membranes to the amniocentesis [13]. The interval was longer when oligohydramnios was present; however, the results reached borderline statistical significance (*p* = 0.08) [13]. In our cohort, we found a longer interval from rupture of membranes to the amniocentesis when oligohydramnios was present. We found a negative correlation between the amniotic fluid index and the interval from rupture of membranes and amniocentesis in the crude and adjusted analysis. The result was controlled for gestational age, which affects the amount of amniotic fluid [31]. This finding appears to be clinically relevant. There is a clear clinical implication, particularly when management of PPROM involved the evaluation of amniotic fluid to rule out microbial invasion of the amniotic cavity and intra-amniotic infection/inflammation. The long interval between rupture of the fetal membranes and amniocentesis appears to be a risk factor for the failure of amniocentesis resulting from low residual amniotic fluid volume. This information could be used for the counseling of pregnant women.

Women with oligohydramnios are at higher risk of cesarean delivery for intrapartum fetal heart rate abnormalities resulting from umbilical cord compression [16], [17], [32], [33]. It has been proposed that fetal heart abnormalities are caused by the loss of the protective effect provided by the amniotic fluid on umbilical cord compression [32], [33]. The finding in this study, that women with oligohydramnios are more likely to undergo cesarean section, is consistent with the findings of other studies.

In this study, we found no difference in the inflammatory and fetal inflammatory response between the groups with and without oligohydramnios. These findings are in conflict with a previous study by Yoon et al., which showed an association between oligohydramnios and a higher intra-amniotic inflammatory response (determined by the amniotic fluid IL-6, IL-1β and tumor necrosis factor-α levels) and the fetal inflammatory response (characterized by the umbilical cord blood IL-6 levels) [13]. Our previous studies showed that the highest intra-amniotic and fetal inflammatory responses in pregnancies characterized by PPROM are elicited when both MIAC and HCA are present [7], [34]. In this study, we found no difference in the presence of women with both MIAC and HCA between the groups with and without oligohydramnios [with oligohydramnios 25% (10/40) vs. without oligohydramnios 21% (7/34); *p* = 0.78; data not shown] [7], [13], [34]. This finding appears to be the reason that no differences in the intra-amniotic and fetal inflammatory responses between the groups with and without oligohydramnios were present in this study. We must take into consideration that there could be population-based differences. These differences could include genetic differences, ethnic differences and differences that might include other environmental and interventional factors. For example, racial/ethnical disparity could play an important role in the inflammatory response. It was shown previously that racial differences exist in immune responses in reproductive tissues ex vivo and in vivo [35]–[38]. It is a highly likely that there are differences between the findings of Yoon et al. and of our study regarding the inflammatory response.

Despite the broad differences among the studies concerning short-term neonatal morbidity, higher rates of respiratory distress syndrome and early-onset sepsis are frequently related to the presence of oligohydramnios [15]–[17]. The results concerning short-term neonatal morbidity are barely comparable because of the different clinical management protocols for PPROM. Oligohydramnios is associated with adverse neonatal outcomes through a shorter latency, particularly in the gestational age-dependent aspects of short-term neonatal morbidity such as respiratory distress syndrome. In this study, we did not find a difference between the presence of oligohydramnios and the prevalence rates of selected aspects of neonatal morbidity. Our results are in concordance with a study by Yoon et al., which investigated only women who delivered within 72 h of amniocentesis [13].

The strength of this study is the short latency between admission and delivery (median 40 h) because of the active management of PPROM, which provides a unique opportunity to combine the data from different compartments (the amniotic fluid, placenta and fetal membranes and umbilical cord blood). There are some limitations to our study. First, the analyses were based on a relatively small sample size, and a type II error might affect our results and could not be ruled out. Second, we did not have information regarding the amount of amniotic fluid before PPROM. We also did not evaluate the dynamics of the amniotic fluid changes during the latency. Third, we did not specifically evaluate the 16S ribosomal RNA in the amniotic fluid, and we could not exclude the presence of uncultivated bacteria in the women without MIAC. Next limitation of this study is that study population did not include women with comorbidities (e.g. autoimmune disease, diabetes mellitus), which can affect inflammatory process. It would be of interest to evaluate the association between residual amount of amniotic fluid and outcomes in this specific subgroup of women with PPROM Nevertheless, it was beyond the scope of this study. The exclusion of women with medical comorbidity from the study prevents us to overemphasize our results on an unselected PPROM population.

The presence of oligohydramnios is not associated with an adverse outcome in actively managed PPROM in singleton pregnancies in the absence of other complications; however, the presence of oligohydramnios is associated with a higher rate of cesarean delivery. Nevertheless, the further larger studies are needed to clarify the association between low amount of residual amniotic fluid and adverse outcomes.
